# A practical guide to the implementation of AI in orthopaedic research—Part 7: Risks, limitations, safety and verification of medical AI systems

**DOI:** 10.1002/jeo2.70247

**Published:** 2025-04-24

**Authors:** Philipp W. Winkler, Bálint Zsidai, Eric Hamrin Senorski, James A. Pruneski, Michael T. Hirschmann, Christophe Ley, Thomas Tischer, Elmar Herbst, Ayoosh Pareek, Volker Musahl, Jacob F. Oeding, Felix C. Oettl, Umile Giuseppe Longo, Kristian Samuelsson, Robert Feldt, ESSKA Artificial Intelligence Working Group

**Affiliations:** ^1^ Department for Orthopaedics and Traumatology Kepler University Hospital GmbH, Johannes Kepler University Linz Linz Austria; ^2^ Department of Orthopaedics, Institute of Clinical Sciences, Sahlgrenska Academy University of Gothenburg Gothenburg Sweden; ^3^ Sahlgrenska Sports Medicine Center Göteborg Sweden; ^4^ Department of Health and Rehabilitation, Institute of Neuroscience and Physiology, Sahlgrenska Academy University of Gothenburg Gothenburg Sweden; ^5^ Department of Orthopaedic Surgery Tripler Army Medical Center Honolulu Hawaii USA; ^6^ Department of Orthopaedic Surgery and Traumatology Kantonsspital Baselland Bruderholz Switzerland; ^7^ University of Basel Basel Switzerland; ^8^ Department of Mathematics University of Luxembourg Esch‐sur‐Alzette Luxembourg; ^9^ Department of Orthopaedic Surgery University Medicine Rostock Rostock Germany; ^10^ Department of Orthopaedic and Trauma Surgery Malteser Waldkrankenhaus Erlangen Erlangen Germany; ^11^ Department of Trauma Hand and Reconstructive Surgery, University Hosptial Muenster Münster Germany; ^12^ Sports Medicine and Shoulder Institute Hospital for Special Surgery New York New York USA; ^13^ Department of Orthopaedic Surgery, UPMC Freddie Fu Sports Medicine Center University of Pittsburgh Pittsburgh Pennsylvania USA; ^14^ Mayo Clinic Alix School of Medicine Mayo Clinic Rochester Minnesota USA; ^15^ Department of Orthopedic Surgery Balgrist University Hospital, University of Zürich Zurich Switzerland; ^16^ Fondazione Policlinico Universitario Campus Bio‐Medico Roma Italy; ^17^ Department of Medicine and Surgery, Research Unit of Orthopaedic and Trauma Surgery Università Campus Bio‐Medico di Roma Roma Italy; ^18^ Department of Orthopaedics Sahlgrenska University Hospital Mölndal Sweden; ^19^ Department of Computer Science and Engineering Chalmers University of Technology Gothenburg Sweden

**Keywords:** AI, certification, digitalization, future, healthcare, risk, safety, standards

## Abstract

Artificial intelligence (AI) has been influencing healthcare and medical research for several years and will likely become indispensable in the near future. AI is intended to support healthcare professionals to make the healthcare system more efficient and ultimately improve patient outcomes. Despite the numerous benefits of AI systems, significant concerns remain. Errors in AI systems can pose serious risks to human health, underscoring the critical need for safety, as well as adherence to ethical and moral standards, before these technologies can be integrated into clinical practice. To address these challenges, the development, certification, and deployment of medical AI systems must adhere to strict and transparent regulations. The European Commission has already established a regulatory framework for AI systems by enacting the European Union Artificial Intelligence Act. This review article, part of an AI learning series, discusses key considerations for medical AI systems such as reliability, accuracy, trustworthiness, lawfulness and legal compliance, ethical and moral alignment, sustainability, and regulatory oversight.

**Level of Evidence:**

Level V.

**Abbreviations**
ACL: anterior cruciate ligament
AI: artificial intelligence
CT: computed tomography
EU: European Union
FDA: Food and Drug Administration
ML: machine learning
MRI: magnetic resonance imaging
PCL: posterior cruciate ligament
PJI: periprosthetic joint infections
TKA: total knee arthroplasty
US: United States
XAI: eXplainable and interpretable AI

## INTRODUCTION

Artificial intelligence (AI) systems have become an integral part of everyday life in making predictions (e.g., risk of disease and weather forecast), performing actions (e.g., autonomous navigation) and generating synthetic data (e.g., creation of texts or images according to the user's requests) [2, 12, 46, 47]. In addition, AI is now impacting the medical world and is contributing to reshaping the current and future healthcare landscape [13, 46]. Since the introduction of AI systems in the 1950s, there has been a tremendous technological progress that is gradually approaching high‐level machine intelligence [10, 40]. High‐level machine intelligence, also referred to as general intelligence or human‐level intelligence, is defined as machines that are able to perform tasks at the same level or better than humans without any assistance. An expert survey on the progress in AI conducted in 2022 indicates a 50% chance to reach high‐level machine intelligence in the year 2059, but progress has been accelerating and a recent update to the survey has revised this prediction forward to 2047 [10, 11].

The basic idea of an AI system is that a statistical model generates output data based on input data. In addition, there is a learning algorithm that allows the system to adjust to become more accurate. AI systems can support clinicians by optimising healthcare processes. Moreover, scientific progress through the use of AI systems can expand knowledge and ultimately help patients [31]. For example, machine learning (ML) models have been suggested as suitable for injury prediction [24]. Pattern recognition models have been implemented to evaluate common medical imaging modalities such as radiographs, magnetic resonance imaging (MRI) and computed tomography (CT) scans [39]. For example, pattern recognition models have already been introduced into clinical practice to detect anterior cruciate ligament (ACL) injuries based on MRI scans [3, 18, 26]. A number of other healthcare topics, such as the prediction of clinical outcomes, skin cancer screening or the evaluation of value‐based metrics to optimise health economic processes (i.e., length of stay, inpatient costs etc.), are already supported by AI systems [17, 20, 28]. Recent generative AI models have also been evaluated for answering medical questions and for automated diagnosis with performance sometimes on par with clinicians [9, 19]. A recent systematic review of AI techniques in orthopaedic disease detection found that most work has focused on fractures, tumours, and deformities while less studies have addressed arthritis and osteoporosis [27].

A thorough understanding of how AI systems function is essential for their safe, effective, and reproducible application in clinical practice. Equally critical are robust regulatory measures to ensure the safety of this rapidly evolving technology. In a survey of nearly 560 AI experts, 5% estimated a significant risk that the long‐term impact of high‐level machine intelligence on humanity could be 'extremely bad' (e.g., human extinction), highlighting the potential dangers posed by AI [10]. Furthermore, 69% of respondents emphasised the need to prioritise AI safety research, calling for 'more' or 'much more' attention to this pressing issue [10]. Safety measures for medical AI systems have remained limited, despite their critical role in ensuring reliable and reproducible use in everyday clinical practice.

This narrative review aimed to address the risks, limitations, and safety measures associated with medical AI systems. It also examined the processes of verification and validation, along with the regulatory frameworks governing the clinical application of AI. Key terms and definitions relevant to this discussion are provided in Tables 1 and 2.

## KEY RISKS AND LIMITATIONS OF AI SYSTEMS

Artificial intelligence has influenced both daily life and clinical practice for several years. However, its use comes with limitations and risks that must be carefully considered. This section explores key challenges in medical AI systems, including ‘distributional shift’, ‘black box decision‐making’ as well as pitfalls of reward‐based AI systems (e.g., reinforcement learning) that are illustrated in detail in this section using fictitious examples. A more complete overview of short‐, medium‐, and long‐term challenges and risks can be found in Challen et al [2].

### Distributional shift

Distributional shift, also known as data‐drift, refers to erroneous predictions of ML models caused by a mismatch between training data and operational data (Table 2) [2, 46]. Such a data mismatch can be triggered by insufficient or biased training data or by the inappropriate use of a trained AI system in an unfamiliar context [2, 44]. Suppose an AI system is developed to predict the risk of periprosthetic joint infections (PJI) in patients undergoing total knee arthroplasty (TKA) and to provide a treatment recommendation (i.e., take an action or take no action) based on the predicted risk (i.e., decision support system). Retrospectively collected healthcare data from a single institution is used to train the AI system. Assume that the selected institution is a private non‐public facility that mainly treats healthy and rather young patients. Once a highly accurate system has been successfully developed, it will be offered to other institutions, maybe also in other countries. Considering that the system is later implemented in a public hospital that treats a high proportion of patients with clinically relevant pre‐existing conditions, the system is likely exposed to a high level of unfamiliar data. This mismatch in training data and operational data may cause erroneous predictions resulting in misleading treatment recommendations. Consequently, actions (e.g., antimicrobial treatment) may be recommended that adversely affect both the individual patient (e.g., allergic reaction) and the healthcare system (e.g., antimicrobial resistance). The above example was fictitious. However, it is worth noting that several ML models for the prevention of PJI after TKA have already been developed [4]. Unfortunately, the majority of these prediction models lack external validation, making them vulnerable to the distributional shift problem and are only valid in the context they were created [4].

### Black box decision‐making

Another major issue of AI systems is called ‘black box decision‐making’ (Table 2). Black box decision‐making means that AI algorithms use methods to produce outputs based on inputs that are not understandable or transparent to humans [46]. In the example of a medical AI system to predict the risk of PJI in patients undergoing TKA, retrospectively collected healthcare data are used as input data. The output from the AI system is the risk of PJI and a treatment recommendation (i.e., take an action or not). However, in most cases it cannot be explained how and why the system got to the specific result, making it difficult to explain or even check for errors. This lack of transparency is a major criticism, raising doubts among users. However, it should be mentioned that human behaviour and decision‐making may also lack transparency. The transparency of AI systems and in particular of decision support systems is essential in order to be trustworthy for users and patients. The importance of trustworthiness of AI systems was emphasised in a statement by the European Commission in 2018. Based on the Ethics Guidelines for Trustworthy AI, a trustworthy AI system should be lawful, ethical, and robust [14]. These three key elements should coexist and orchestrate in a trustworthy AI system to avoid unintended harmful behaviour. Extensive research and development efforts are also focused on advancing eXplainable and interpretable AI (XAI) systems, in order to mitigate the black‐box nature of AI systems [23]. Interpretable AI aims to create models that are inherently transparent and understandable, such as methods that identify the provably optimal scoring system [41]. Explainable AI, on the other hand, involves techniques that illuminate the factors influencing a model's specific recommendations, providing clearer insights into its decision‐making processes.

### Reinforcement learning

Reinforcement learning is a promising key concept in ML. In reinforcement learning, the AI system aims to perform actions in order to maximise its reward (to be defined in the given context) in a dynamic environment. Based on the actions performed, the system obtains positive (i.e., reward) or negative (i.e., penalty) feedback. Positive feedback will reinforce the system to perform similar actions to gain more reward [45]. AlphaFold is a popular example of an AI system developed by Google DeepMind (London, England) using reinforcement learning. AlphaFold is intended to predict the three‐dimensional protein shape based on the amino acid sequence and helps to solve the protein‐folding problem. Back to the above example: assume that an improved version of the AI system for predicting PJI in TKA is now able to provide a detailed antimicrobial treatment regime for patients at high risk of PJI. A reward is given to the system when a treatment recommendation is made that covers the corresponding antimicrobial profile. To avoid negative feedback (i.e., penalties), the AI system may recommend comprehensive antimicrobial coverage for every patient in order to cover most pathogens (i.e., overtreatment), even if antimicrobial treatment is not necessary for some patients. Consequently, a differentiated treatment recommendation will be lost, increasing the risk developing antimicrobial resistance in the long‐term.

While AI systems can be designed to achieve greater accuracy, precision, explainability, or interpretability, it is crucial to recognise that their output quality is directly dependent on the quality of the input data and the environment in which they operate. False predictions often stem from insufficient, unaligned, or poor‐quality training data, a challenge commonly referred to as the 'garbage‐in‐garbage‐out' principle [44]. This issue is particularly relevant for AI systems relying on retrospective data and must be carefully addressed in their development and application.

## SAFETY MEASURES

In view of the aforementioned risks and limitations of AI systems, safety measures for medical AI systems are becoming increasingly important. Reliability, accuracy, validity, trustworthiness, lawfulness, ethical and moral conformity, sustainability, as well as regulatory oversight are emerging topics in medical AI systems (Table 2). Insufficient safety measures and regulatory actions can lead to systemic errors arising from AI systems. Such systemic errors are difficult to identify owing to circumstances like the black‐box phenomenon or the distributional shift problem and can ultimately cause harm to the patients [5].

AI safety measures are intended to accurately detect unintended behaviour of AI systems and take appropriate measures to avoid adverse effects. Key concepts for safe and reliable operation of AI systems cover the domains specification, robustness, and assurance (Table 1) [30, 32].

**Table 1:** Key concepts in AI safety.

Term	Definition	Aim
Specification	Purpose of the system	Ensure that the AI system operates as intended by the developer.
Robustness	Resistance to perturbation	Ensure that the AI system continues to operate within predefined safety limits, even in unfamiliar system conditions.
Assurance	Monitoring and controlling	Ensure that the AI system is interpretable and understandable for users.

Abbreviation: AI, artificial intelligence.

### Specification

An important task when developing an AI system is to precisely define what the AI system is intended to do. The precise definition of how the AI system should operate is called *specification* and is a key element for the designer of an AI system. In particular, reward‐driven AI systems are prone to unintended behaviour in case of insufficient specification [35]. Three levels of specification are typically identified: ideal specification, design specification and revealed specification. While the ideal specification represents the function of the system desired by the designer, the design specification represents the function that has been implemented in the AI system. Lastly, the revealed specification represents how the AI system ultimately operates. Challenging tasks, complex operating environments, or unpredictable actions of the AI system may cause a mismatch between the revealed specification and the ideal specification, eventually leading to unintended and potentially harmful events [35].

### Robustness

Once the AI system has been trained properly, it can work as intended. Unfortunately, attacks such as data poisoning or adversarial examples (Table 2) may lead to misleading prediction of trained AI systems. To avoid system failures caused by adversarial attacks, an AI system must be resistant to perturbations and irregularities, which is referred to as *robustness*. Predictive uncertainty estimates have been introduced to indicate the level of uncertainty of the AI system concerning the correctness of its prediction [34]. Such estimates can help users to assess the reliability of an AI system's prediction.

**Table 2:** Terms and definitions.

Term	Definition
Accuracy	The degree of correctness that an AI system generates correct outputs or predictions based on the given inputs.
Adversarial example	Adversarial examples are inputs for AI systems that intentionally contain minor errors. This leads to misinterpretations and ultimately to unintended behaviour of the AI system. In this way, the robustness of AI systems should be improved.
Black box decision‐making	Output/decision of an AI system based on patterns and correlations of big sets of training data. However, the underlying rationale for the output/decision is unknown.
Data poisoning	Poisoning/contaminating the training data to increase errors in the output of AI systems.
Distributional shift	Erroneous predictions of AI systems caused by a mismatch between training data and operational data.
Lawfulness	AI systems must comply with national and international legislation.
Reliability	Consistency and stability of an AI system over time and in different environments.
Trustworthiness	An AI system that operates lawful (respects laws and regulations), ethical (respects ethical principles and values), and robust.

Abbreviation: AI, artificial intelligence.

### Assurance

Expected and unexpected behaviour of AI systems should be understandable and interpretable for human operators. Given that actions of medical AI systems may have a serious impact on human lives, it is crucial that the system's behaviour is understood by users and therefore trustworthy. The interpretability of modern AI systems can be seen as a means of *assurance* that has become increasingly important in recent years. Deep neural networks, for example, are highly developed ML models that use a large number of hierarchically organised layers and a complex connection of a variety of parameters to generate output data from the input data. Such networks contain so‐called hidden layers that create complex and deep connections. Consequently, it is often difficult to explain how the output data comes about. This is why it is often referred to as a black box phenomenon [13, 33].

**Figure 1:**
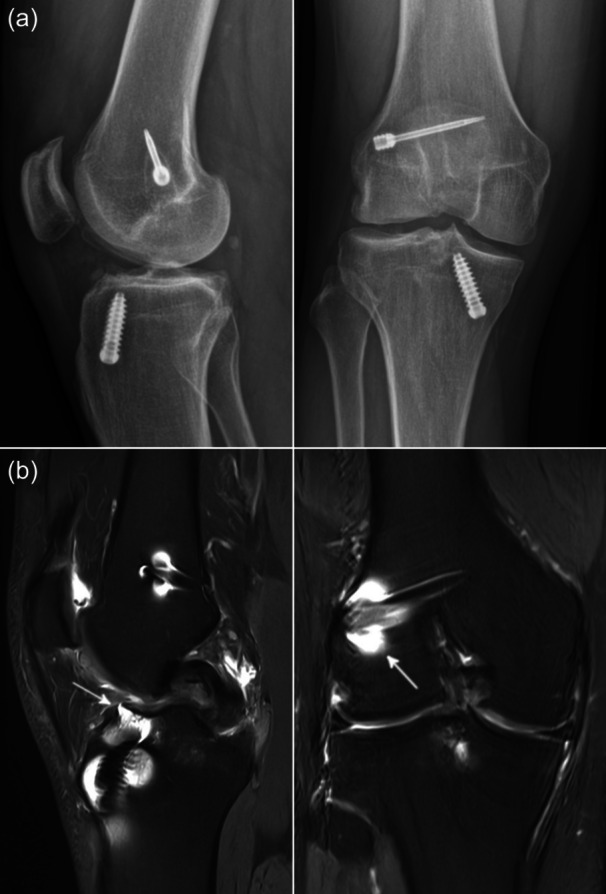
Unintended perturbations in image recognition. Anteroposterior and lateral radiographs (a) and proton density weighted coronal and sagittal magnetic resonance imaging (MRI) scans (b) of a right knee with failed transtibial anterior cruciate ligament reconstruction. Note the tibial and femoral implants for graft fixation and the corresponding metallic MRI artifacts (arrows).

The following example is intended to demonstrate the importance of the key concepts in AI safety (i.e., specification, robustness and assurance) in a clinical scenario. Recently, a number of studies have been published in which AI‐based models for the detection of ACL injuries have been developed based on MRI scans [3, 18, 26, 39]. A diagnostic sensitivity and specificity of 87%–97% and 86%–100% have been described [3, 26, 39]. The goal of the AI system (i.e., detection of an ACL injury) must be precisely and clearly defined (i.e., specification). For example, if the AI system is rewarded by detecting increased signal intensity caused by a ligament injury, it may incorrectly classify a posterior cruciate ligament (PCL) injury as an ACL injury. Furthermore, an AI system must be resistant to unintended perturbations (i.e., robustness). For example, when detecting ACL injuries, metallic artifacts from implants from previous surgical procedures may cause an unfamiliar appearance of the ACL (Figure 1). In such cases, the AI system should recognise the abnormal situation and resort to a safe fallback strategy such as alerting human review. Note that triggering human review too frequently can lead to a well‐known phenomenon called alarm fatigue [44]. Physicians aim not only for the correct diagnosis, but also for the diagnostic pathway. Therefore, assurance of an AI system is important to trust the algorithm. Accordingly, it would be of major interest that an AI‐based ACL injury detection tool also indicates the diagnostic criteria monitored to diagnose an ACL injury.

The potential risks and safety measures for the use of AI systems mentioned in this article cover relevant parts to orthopaedic surgeons and scientists. More detailed information can be found at specialised institutions such as the Center for Security and Emerging Technology (CSET; https://cset.georgetown.edu) or the Center for AI Safety (https://www.safe.ai).

## REGULATION OF AI SYSTEMS

The use of AI systems raises critical issues, including ethical considerations, socio‐economic impacts, data security and data protection, all of which require regulatory oversight to uphold ethical standards and safeguard sensitive information. While regulatory oversight of AI was once a relatively minor concern, it has gained significant importance in recent years, driven by heightened political activity and a growing recognition of its necessity. In October 2023, the president of the United States signed the first AI executive order to control the use of AI systems [38]. This executive order was issued to set standards for AI safety and security, enhance the transparency of AI systems, and protect the privacy and civil rights of the American population [38]. In addition, an AI safety summit was hosted in the UK with representatives from around the world and leading technology companies [16]. The European Union (EU) Artificial Intelligence Act was first presented in April 2021 by the European Commission, agreed by the European Parliament in December 2023, and recently, in August 2024, went into force (https://commission.europa.eu/news/ai-act-enters-force-2024-08-01_en). The EU AI Act represents a safety framework with requirements and obligations for the development and use of AI systems within the EU [21].

The safety policy introduced by the EU AI Act sets requirements for market entrance and certification of AI systems. This includes, for example, a mandatory CE marking process for high‐risk AI systems, which guarantees health, safety, and environmental standards determined by the EU. The EU AI Act follows the Ethics Guidelines for Trustworthy AI proclaimed by the European Commission, requiring AI systems to be lawful, ethical and robust while ensuring democratic values, human rights, and the rule of law [14].

**Figure 2:**
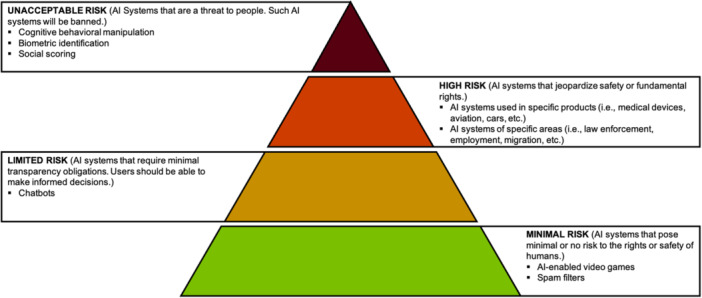
Risk categories proposed by the European Union Artificial Intelligence Act.

In addition, a key interest for the EU is that AI systems are continuously monitored by humans and not by 'automated machines'. A risk‐based approach for regulatory oversight of well‐defined AI systems has been announced by the European Commission. Accordingly, the EU AI Act defines four risk categories, each with specific requirements, to guarantee safety and avoid adverse events of all types of AI systems (Figure 2) [8, 21]. A similar risk‐based approach has also been employed by the US Food and Drug Administration (FDA) to classify and regulate AI systems. Based on the risk profile, the FDA classifies medical devices into three different risk categories. Most AI systems such as image recognition systems etc. belong to Class II (moderate risk). However, AI systems used for critical medical decisions belong to Class III (high risk) [25].

The agreement reached in December 2023 on the world's first comprehensive AI law has been officially adopted to by the Parliament and Council and is expected to ultimately become EU law during 2026 (https://commission.europa.eu/news/ai-act-enters-force-2024-08-01_en) [8]. This is a major step forward in the regulatory oversight of AI systems within the EU, but it will likely take time before a practice for how to interpret the law develops.

## VERIFICATION AND VALIDATION OF AI SYSTEMS

Given the significant impact of diagnostic and therapeutic actions on human health, the accuracy, validity, reliability and trustworthiness of medical AI systems is often critically questioned. Reasons for this uncertainty have already been explained in detail and include insufficient data quality, misspecification, lack of transparency and many more. Therefore, the correctness and dependability of medical AI systems is essential to avoid harm to patients. Verification and validation of AI systems, also known as verified AI, is a process that uses formal verification techniques to ensure the correctness and reliability of AI systems [1, 36]. A verified AI system requires strict goals to be specified in the design phase. The actual behaviour of the system is then verified and validated using mathematical models. In this way, erroneous actions and system bugs can be detected and eliminated [1]. However, as the complexity of the data and the considerations that an AI system needs to take increases, it may not be practical or cost‐efficient to use formal verification techniques and more statistical approaches may be warranted [15].

### Validation

In general, internal validation has to be distinguished from external validation. Internal validation is performed with unknown data originating from the same dataset that was used to train the AI system's algorithm. Note that basic characteristics (e.g., ethnicity, time period, hospital etc.) are identical between the training dataset and the internal validation data set. In contrast, external validation is performed using external data that are new to the system [7, 37]. Despite the tremendous increase of medical AI systems over the past years, recent studies show that only a minority of AI systems undergo external validation [4, 22, 29]. However, the importance of external validation has been emphasised in a recent study, showing that the performance of AI systems strongly depends on the dataset (internal validation data set versus external validation data set) [29]. One recent systematic review investigating deep learning studies that performed external validation found that most models demonstrated worse performance on the external dataset, with almost 25% of studies reporting a substantial performance decrease defined as greater than 0.10 on the unit scale [43].

### Process of verification and validation

Verification and validation of AI systems comprises several steps. First, the intended purpose of the AI system must be clearly specified so that the goal of the verification and validation process is also unambiguous. Next, a data set for internal and external validation must be defined. Preferably, the validation data set consists of unbiased “real word” data. This key step should be done diligently, as the quality of the data affects the generalisability of the AI system. The verification and validation process should be performed in a controlled and transparent fashion and the results need to be reported in detail. Strengths and weaknesses of the system should be recognised and causes of errors identified. This allows the system to be optimised, reducing operational risks. Finally, the performance of the verification and validation process should be reviewed by experts and the reliability and quality of the verification and validation process should be reported. Standards for how to evaluate and report on the performance of clinical prediction models can also be useful and help ensure proper verification and validation practices [6]. Given that a very large number of performance measures for AI classification models have been proposed, recent guidance can be used to select an essential and safe set to use [42].

## CONCLUSION

Artificial intelligence is rapidly evolving and gradually finding its way into daily clinical practice. Scientists and orthopaedic surgeons should be acquainted with the basic principles of AI and most common risks and limitations (e.g. ‘distributional shift’, ‘black box decision‐making’, and reinforcement learning) to ensure a safe and reproducible application in daily clinical practice. AI systems should complement and support clinicians and scientists but not replace them. Individualised and value‐based treatment supported by AI systems helps orthopedic surgeons to focus on patients and ultimately improve patient outcomes. However, medical AI systems must comply with regulatory requirements and operate in a reliable, lawful, and trustworthy manner.

## AUTHOR CONTRIBUTIONS

All listed authors have contributed substantially to this work: Philipp W. Winkler, Bálint Zsidai, Felix C. Oettl, and Eric Hamrin Senorski performed literature review, and primary manuscript preparation. Editing and final manuscript review and preparation was performed by Philipp W. Winkler, James A. Pruneski, Michael T. Hirschmann, Christophe Ley, Thomas Tischer, Elmar Herbst, Ayoosh Pareek, Volker Musahl, Jacob F. Oeding, Umile Giuseppe Longo, Robert Feldt, and Kristian Samuelsson. All authors read and approved the final manuscript.

## CONFLICT OF INTEREST STATEMENT

PWW reports working as a web editor for Knee Surgery, Sports Traumatology, Arthroscopy (KSSTA). EHS reports working as associate editor for the Journal of Orthopaedics & Sports Physical Therapy. EH reports working as deputy editor‐in‐chief of of Knee Surgery, Sports Traumatology, Arthroscopy (KSSTA). VM reports educational grants, consulting fees, and speaking fees from Smith & Nephew plc, educational grants from Arthrex, is a board member of the International Society of Arthroscopy, Knee Surgery and Orthopaedic Sports Medicine (ISAKOS), and deputy editor‐in‐chief of Knee Surgery, Sports Traumatology, Arthroscopy (KSSTA). MTH reports educational grants, consulting fees, and speaking fees from DepuySynthes, Symbios, Medacta and editor‐in‐chief of Knee Surgery, Sports Traumatology, Arthroscopy (KSSTA). KS is a member of the Board of Directors of Getinge AB (publ) and medtech advisor to Carl Bennet AB. JFO is a consultant for Kaliber.ai. RF is CEO of Accelerandium AB.

## ETHICS STATEMENT

Not applicable.

## Data Availability

Data sharing not applicable to this article as no datasets were generated or analysed during the current study.
